# Case Report: Late repeat pancreatic irreversible electroporation in the long-term multidisciplinary management of unresectable pancreatic ductal adenocarcinoma

**DOI:** 10.3389/fonc.2026.1944908

**Published:** 2026-09-09

**Authors:** Yingjie Wang, Qingmin He, Hao Li, Tao Guo, Guizhi Wang, Xiang Li, Meng Fei, Yanjun Chen

**Affiliations:** 1 Department of Hepatobiliary and Pancreatic Surgery, The Fifth Affiliated Hospital of Zhengzhou University, Zhengzhou, China; 2 Henan Key Laboratory of Helicobacter pylori, Microbiota and Gastrointestinal Cancer, Marshall Medical Research Center, The Fifth Affiliated Hospital of Zhengzhou University, Zhengzhou, China; 3 Department of Pediatric Intensive Care, The Fifth Affiliated Hospital of Zhengzhou University, Zhengzhou, China; 4 Department of Pathology, The Fifth Affiliated Hospital of Zhengzhou University, Zhengzhou, China

**Keywords:** case report, irreversible electroporation, liver metastasis, locally advanced pancreatic cancer, long-term survival, multidisciplinary management, pancreatic ductal adenocarcinoma, repeat pancreatic irreversible electroporation

## Abstract

Unresectable locally advanced pancreatic ductal adenocarcinoma (PDAC) has a poor prognosis, particularly after distant metastasis. Irreversible electroporation (IRE) is a nonthermal ablative technique used for pancreatic tumors adjacent to major vessels, but evidence on late repeat pancreatic IRE remains limited. We report a 62-year-old man who presented with obstructive jaundice. Multidisciplinary evaluation classified the tumor as unresectable locally advanced PDAC involving the superior mesenteric vein-portal vein confluence. In January 2021, he underwent pancreatic IRE with biliary bypass, followed by sequential systemic therapy. Liver metastases developed approximately 10 months later and were treated locally, after which he continued multimodal therapy. In April 2025, gastroscopy revealed deformity and luminal narrowing of the duodenal bulb, accompanied by gastric retention, for which laparoscopic gastrojejunostomy was performed. In late 2025, a marked rise in carbohydrate antigen 19-9 (CA19-9) and tumor progression prompted multidisciplinary reassessment. Nearly 5 years after the initial procedure, he underwent repeat pancreatic IRE combined with radiofrequency ablation of a hepatic metastasis. During subsequent multimodal management, CA19–9 decreased from 12,280.0 to 291.4 U/mL. As of March 2026, he remained alive with disease, with an overall survival exceeding 62 months. This case demonstrates the technical feasibility of repeat pancreatic IRE after a prolonged interval and supports its potential role as an individualized local treatment within long-term multidisciplinary management.

## 1 Introduction

Pancreatic ductal adenocarcinoma (PDAC) is one of the digestive system malignancies associated with the poorest prognosis (1). Locally advanced pancreatic cancer (LAPC) is often considered unresectable because of major vascular involvement (2). Although systemic therapy remains the foundation of treatment, local pancreatic disease may still cause biliary or gastrointestinal obstruction, pain, and vascular complications. Integrating local and systemic treatments under multidisciplinary team (MDT) guidance is therefore important in the long-term management of unresectable LAPC.

Irreversible electroporation (IRE) is a nonthermal ablation technique that induces cell death through irreversible changes in cell membrane permeability. Because its primary ablative effect does not rely on sustained high temperatures, IRE may reduce heat-related tissue injury and allow relative preservation of the structural framework of adjacent blood vessels and ducts, supporting its use for pancreatic lesions near critical structures. In carefully selected patients, IRE may provide local treatment within an MDT framework rather than independent curative therapy (3). Evidence regarding repeat pancreatic IRE remains limited, with cohort studies describing only small numbers of patients retreated for residual disease, local recurrence, or local progression after initial IRE (4–7) and other reports documenting isolated cases (8, 9). Experience with retreatment several years after initial IRE is particularly scarce.

We report a 62-year-old man who presented with obstructive jaundice and whose tumor was clinically assessed by MDT in 2021 as unresectable LAPC involving the superior mesenteric vein-portal vein confluence. Following initial pancreatic IRE and sequential multimodal treatment, liver metastases developed. Nearly 5 years later, he underwent repeat pancreatic IRE combined with radiofrequency ablation of a hepatic metastasis and remained alive with disease for more than 62 months. Based on this case and the available literature, we discuss the clinical significance and limitations of late repeat pancreatic IRE in the long-term MDT management of initially unresectable LAPC.

## 2 Case description

### 2.1 Initial presentation and diagnostic assessment

A 62-year-old man was admitted to The Fifth Affiliated Hospital of Zhengzhou University on January 6, 2021, with a history of progressive jaundice lasting more than 1 month. Associated symptoms included decreased appetite, dark urine, pale stools, periumbilical pain, and an unintentional weight loss of approximately 10 kg. His medical history included hypertension, type 2 diabetes mellitus, and lacunar cerebral infarction. He reported no known family history of malignancy. At initial presentation, he had an Eastern Cooperative Oncology Group (ECOG) performance status of 2. Laboratory evaluation on admission revealed marked cholestasis and hepatocellular injury: TBIL, 93.0 μmol/L; DBIL, 58.6 μmol/L; alanine aminotransferase (ALT), 267 U/L; aspartate aminotransferase (AST), 154 U/L; alkaline phosphatase (ALP), 612 U/L; and gamma-glutamyl transferase (GGT), 2,211 U/L. Serum CA19–9 was elevated to 317.16 U/mL.

**Figure 1:**
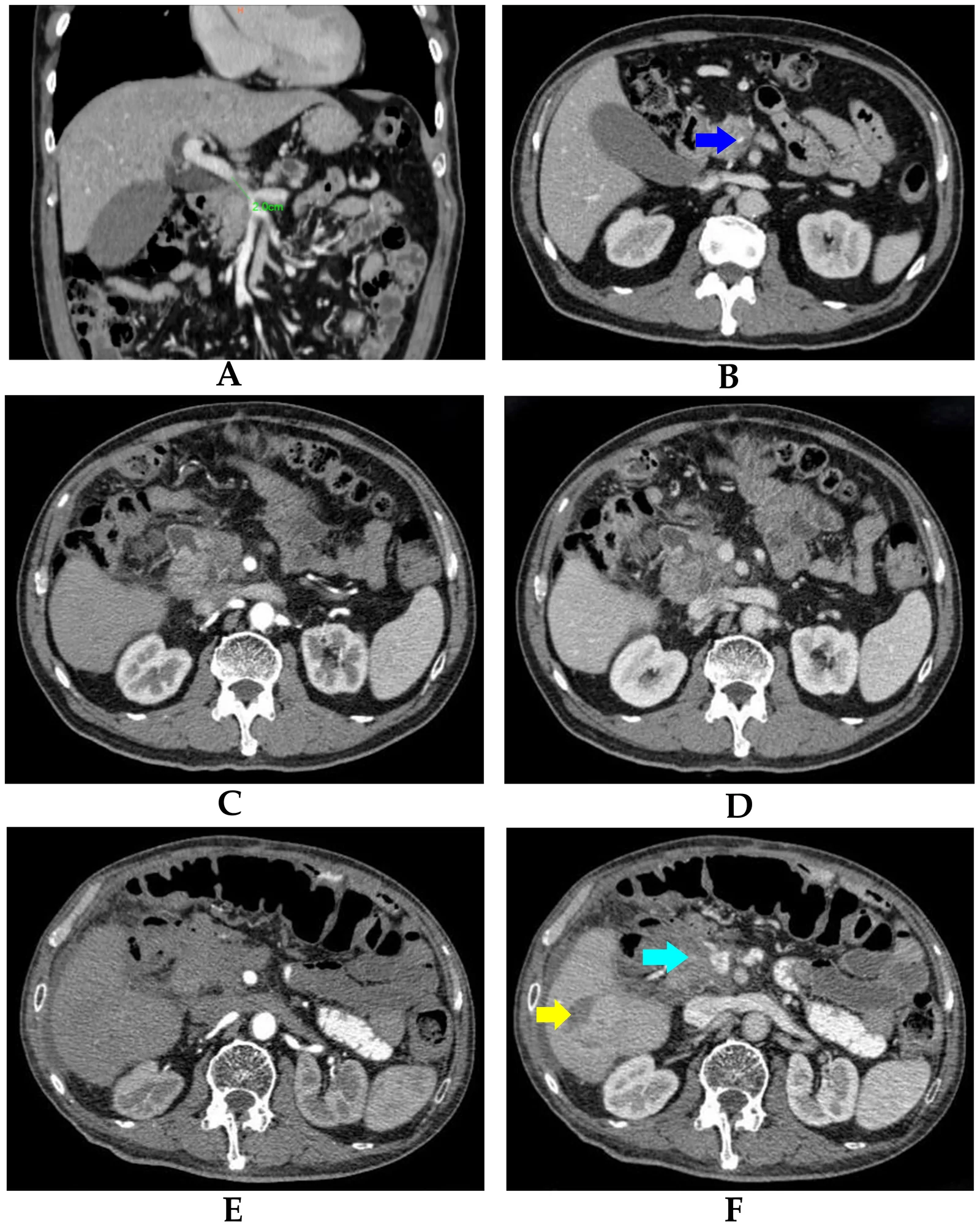
Longitudinal contrast-enhanced CT findings across the treatment course. **(A, B)** Contrast-enhanced CT images obtained before the initial IRE procedure in January 2021. **(A)** Coronal image showing approximately 2.0 cm of craniocaudal venous involvement at the superior mesenteric vein–portal vein (SMV–PV) confluence. **(B)** Axial image showing tumor involvement and marked narrowing of the SMV–PV confluence (blue arrow). **(C, D)** Arterial- and portal venous-phase contrast-enhanced CT images obtained during follow-up in August 2023, showing that the local pancreatic lesion remained relatively stable. **(E, F)** Arterial- and portal venous-phase contrast-enhanced CT images obtained in January 2026 after repeat pancreatic IRE and RFA of the hepatic metastasis. In **(F)**, the cyan arrow indicates the pancreatic IRE-treated area, and the yellow arrow indicates the hepatic RFA-treated area.

Abdominal computed tomography (CT) and ultrasonography showed dilatation of the intrahepatic and extrahepatic bile ducts and pancreatic duct, with gallbladder enlargement. Contrast-enhanced CT and magnetic resonance imaging revealed an irregular, poorly defined mass in the pancreatic head and uncinate process involving the superior mesenteric vein-portal vein confluence. Tumor-vein contact exceeded 180°, with marked narrowing and approximately 2.0 cm of craniocaudal involvement (**Figures 1A, B**). No definite major arterial involvement or distant metastases were identified. Based on contemporaneous MDT review, the tumor was clinically considered unresectable LAPC because the involved venous segment was considered unsuitable for safe resection and reconstruction, limiting the feasibility of R0 resection. Given obstructive jaundice and unresectability, the MDT recommended IRE with biliary bypass for obstruction relief and local tumor control. Needle biopsy was planned during the same procedure to establish histopathology and guide systemic treatment.

### 2.2 Initial pancreatic IRE and pathological diagnosis

On January 13, 2021, the patient underwent surgical exploration and local treatment. Intraoperatively, the gallbladder was markedly distended, and cholestatic changes were observed in the liver. Palpation during surgical exploration identified a firm, fixed, and poorly mobile mass measuring approximately 4.0 × 3.5 cm in the pancreatic head. Consistent with the preoperative imaging findings, extensive local vascular involvement was confirmed, and curative resection was deemed infeasible. A needle biopsy specimen was first obtained directly from the pancreatic lesion under intraoperative ultrasound guidance, after which pancreatic IRE was performed.

Three monopolar electrodes were then placed under continued ultrasound guidance, with 1.5-cm exposed tips and approximately 1.2-1.5-cm inter-electrode spacing. The initial electric field strength and pulse width were 1500 V/cm and 70 μs, respectively. Each pair first received 10 test pulses, yielding currents of 25–35 A; 70 treatment pulses were then delivered. During treatment, current increased by 10–15 A to 40–50 A; based on real-time feedback, an additional 70- or 90-pulse series was delivered as required. All pulses were delivered under electrocardiographic synchronization and adequate neuromuscular blockade. To cover the planned treatment area, the electrodes were repositioned nine times under real-time ultrasound guidance, creating overlapping treatment zones. Active ablation and total IRE procedure times were approximately 2 and 3 h, respectively. No arrhythmias, significant hemodynamic instability, or other procedure-related adverse events occurred. After IRE, cholecystectomy, biliary-enteric anastomosis, and enteroenterostomy were performed.

**Figure 2:**
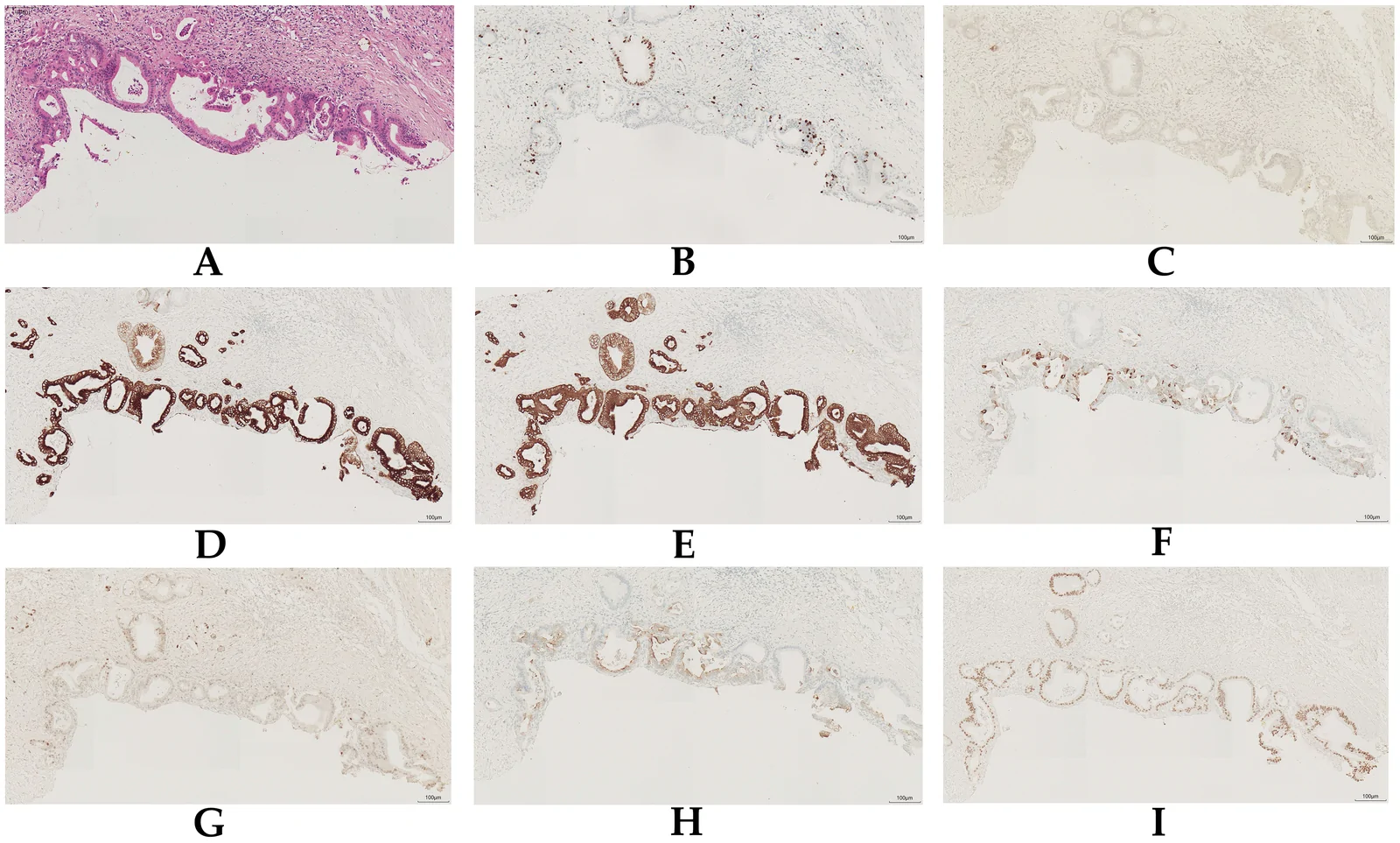
Histopathological and immunohistochemical findings of the pancreatic lesion. **(A)** H&E staining shows sparse infiltrating atypical glands with marked stromal fibrosis. **(B–I)** Immunohistochemical staining shows, respectively, a Ki-67 proliferation index of approximately 15%, loss of SMAD4 expression, CK7 positivity, CK19 positivity, focal CK20 positivity, a heterogeneous wild-type p53 expression pattern with nuclear staining in approximately 40% of tumor cells, CEA positivity, and focal CDX2 positivity. Scale bars represent 100 μm.

The patient recovered uneventfully, with no documented perioperative complications. TBIL and DBIL declined from 93.0 and 58.6 μmol/L on January 6 to 35.1 and 18.0 μmol/L on postoperative day 2, respectively. By January 30, 2021, TBIL had further declined to 19.3 μmol/L, indicating marked relief of biliary obstruction following IRE with biliary bypass. Serum amylase was 43 U/L on postoperative day 2, without post-IRE hyperamylasemia. Histopathological examination of the pancreatic biopsy specimen revealed sparse infiltrating atypical glands within a markedly fibrotic stroma. Immunohistochemical analysis showed positive expression of CEA, CK19, and CK7. The Ki-67 proliferation index was approximately 15%, p53 showed a heterogeneous wild-type expression pattern, with nuclear positivity in approximately 40% of tumor cells, and loss of SMAD4 expression was observed. Although CK20 and CDX2 showed focal positivity, diffuse staining suggestive of an intestinal primary tumor was absent. Testing for MMR protein expression, MSI, TMB, KRAS, and germline BRCA1/2 and PALB2 was not performed. Based on the histopathological features, immunophenotypic profile, tumor location, and radiological findings, the diagnosis was adenocarcinoma of pancreatobiliary origin, clinically consistent with PDAC (**Figure 2**) (10).

### 2.3 Multimodal treatment and long-term follow-up

On April 13, 2021, the patient began chemotherapy with nab-paclitaxel plus gemcitabine (11). In June, CA19–9 increased to >700 U/mL, and imaging suggested possible residual disease or local progression within the pancreatic head IRE-treated area. Chemotherapy was discontinued after five cycles because of poor tolerance. A trial of local radiotherapy yielded no clear response. Following further MDT reassessment, camrelizumab, a programmed cell death protein 1 (PD-1) inhibitor, was initiated on August 28, 2021, at 200 mg intravenously approximately every 2 weeks. During subsequent follow-up, the pancreatic head lesion showed no obvious further progression, and CA19–9 decreased to 369.86 U/mL.

In November 2021, follow-up CT revealed multiple new hypoenhancing liver lesions, with measurable lesions in segments V (2.0 × 1.6 cm) and II (1.8 × 1.5 cm), while the pancreatic head lesion remained stable. Based on clinical history and multimodal imaging, these lesions were clinically diagnosed as metastases and treated with laparoscopic ultrasound-guided radiofrequency ablation (RFA) following MDT reassessment. Subsequent imaging showed no persistent activity in the treated regions, and CA19–9 decreased to 356.78 U/mL approximately 1 month later. On February 16, 2022, he began modified FOLFIRINOX (mFOLFIRINOX) (12) but discontinued it after three cycles because of poor tolerance. Camrelizumab was continued, with anlotinib added on April 10, 2022, at 8 mg orally once daily on days 1–14 of each 21-day cycle. Follow-up imaging in June 2022 showed that the irregular hypodense pancreatic head lesion remained largely unchanged. From May to August 2023, CA19–9 remained relatively stable at 80.88-105.90 U/mL, and contrast-enhanced CT in August 2023 showed that the pancreatic lesion remained stable (**Figures 1C, D**).

Treatment with camrelizumab and anlotinib continued until January 29, 2024. On March 5, 2024, CA19–9 was 361.5 U/mL. Given concern about diminishing efficacy after prolonged camrelizumab treatment, adebrelimab was initiated after MDT evaluation at 1,200 mg intravenously every 3 weeks. During continued clinical and imaging follow-up, gastroscopy in April 2025 revealed deformity and luminal narrowing of the duodenal bulb with gastric retention. The patient underwent laparoscopic gastrojejunostomy on April 22, after which symptoms improved. Adebrelimab was continued, and no clear tumor progression was observed until September 2025. From September to October 2025, disease progression was observed, with CA19–9 increasing from 643.8 U/mL on September 10 to 12,280.0 U/mL on October 22. In the absence of jaundice and with TBIL remaining normal at 4.2-5.8 μmol/L, the CA19–9 increase was considered more likely to reflect tumor progression than biliary obstruction (13, 14). PET/CT showed heterogeneously increased uptake in the pancreatic head, raising concern for residual or progressive tumor. Ultrasonography showed a 34 × 36 mm hypoechoic lesion in the pancreatic head closely associated with the superior mesenteric vein and a 22 × 13 mm hypoechoic lesion in the pancreatic body. PET/CT also identified a new mildly FDG-avid low-attenuation lesion in hepatic segment VI; ultrasonography showed a corresponding 36 × 43 × 55 mm heterogeneous echogenic lesion. At MDT reassessment, the patient had an ECOG performance status of 1, and the pancreatic and hepatic lesions were considered suitable for further local treatment.

### 2.4 Repeat pancreatic IRE and outcomes

On November 18, 2025, the patient underwent repeat pancreatic IRE and RFA of the hepatic lesion. Under ultrasound guidance, the IRE electrodes were positioned while avoiding the portal vein, celiac axis, superior mesenteric vessels, and adjacent bowel. Each electrode pair received 10 test pulses followed by 90 treatment pulses with a pulse width of 90 μs; all other principal settings were unchanged from the initial IRE, including an electric field strength of 1500 V/cm, a 1.5-cm exposure length, and 1.2-1.5-cm electrode spacing. Pulses were delivered under electrocardiographic synchronization and adequate neuromuscular blockade. Guided by lesion distribution and adjacent vascular anatomy, multi-electrode placement and sequential repositioning created five overlapping treatment zones covering the planned pancreatic treatment area. Four RFA electrodes were then placed in the 36 × 43 × 55 mm hepatic segment VI lesion and activated in pairs over two ablation cycles. Intraoperative ultrasound confirmed coverage of the planned treatment areas; no active bleeding occurred.

**Figure 3:**
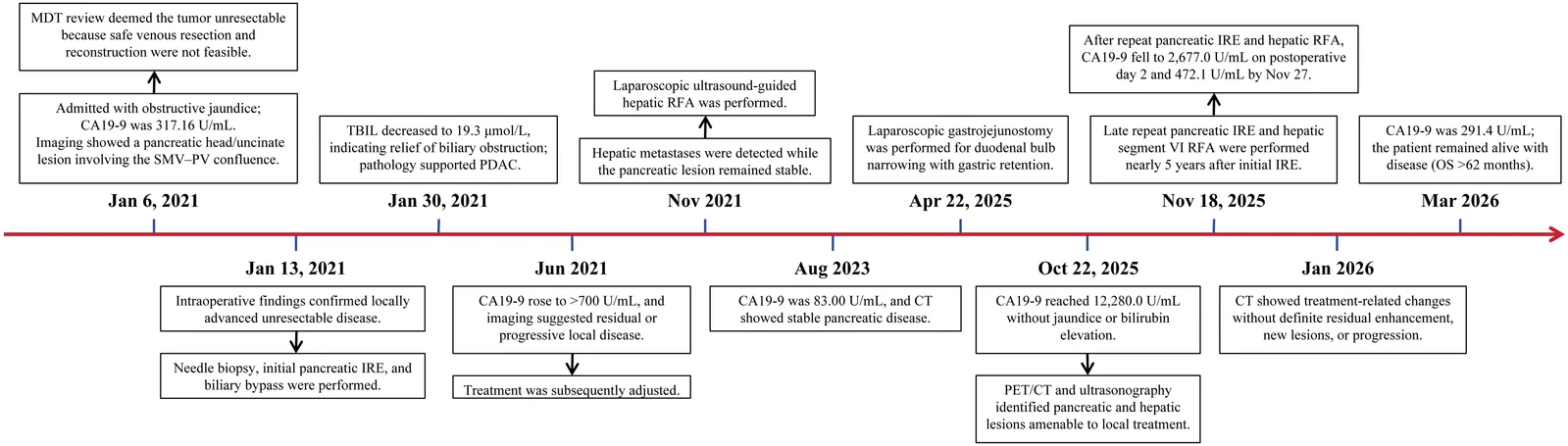
Timeline of the patient’s clinical course, treatment, and major outcomes from the initial presentation on January 6, 2021, to the last follow-up in March 2026.

The postoperative course was uncomplicated, and serum amylase and lipase on postoperative day 1 were 40 and 7 U/L, respectively, with neither elevated. On October 22, 2025, the preoperative CA19–9 level was 12,280.0 U/mL. After repeat pancreatic IRE and hepatic RFA, CA19–9 fell to 2,677.0 U/mL on postoperative day 2 and 472.1 U/mL on November 27. Adebrelimab had last been administered on September 11 and was withheld for surgery; it was resumed on December 5, with no intervening systemic antitumor therapy. Contrast-enhanced CT in January 2026 showed treatment-related changes at the pancreatic IRE and hepatic RFA sites, with no definite residual enhancement, new lesions, or radiologic progression (**Figures 1E, F**). After adebrelimab was resumed, CA19–9 further declined to 291.4 U/mL on March 5, 2026. At the last follow-up in late March 2026, approximately 4 months after repeat treatment, the patient remained alive with disease and had an ECOG performance status of 1; overall survival exceeded 62 months. The clinical course and treatment timeline are summarized in **Figure 3**.

## 3 Discussion

Systemic therapy remains the foundation of treatment for LAPC and metastatic pancreatic cancer, with FOLFIRINOX and gemcitabine plus nab-paclitaxel widely used (11, 15); chemoradiotherapy may also be incorporated in selected patients (16). However, persistent local tumor burden, tumor-related symptoms, or limited progression despite systemic treatment may compromise biliary and gastrointestinal patency, impair pain control, and increase the risk of further vascular involvement. Selective incorporation of local therapies therefore remains an important component of multidisciplinary management (17). Because its primary ablative effect does not depend on sustained high temperatures, IRE may reduce heat-sink effects and heat-related injury, with the potential for relative preservation of the structural framework of adjacent vessels and ducts (18–20). However, pancreatitis and vascular, biliary, and gastrointestinal complications remain possible (7, 21, 22). Clinical studies and reviews suggest that IRE may serve as a component of multimodal treatment in selected patients (23–25). Accordingly, IRE is more appropriately considered a local-control modality within a multimodal framework rather than an independent curative treatment (26). Within this framework, evidence regarding repeat pancreatic IRE remains particularly limited.

To contextualize the present case, we conducted a structured narrative review by searching PubMed and Web of Science Core Collection from inception to June 30, 2026. Search strategies combined pancreatic terms (“pancreas” and “pancreatic”), IRE (“irreversible electroporation”), and retreatment or local-failure terms (“repeat,” “repeated,” “second,” “retreatment,” “residual tumor,” “local recurrence,” and “local progression”). Reference lists of relevant reports were also screened for potentially eligible publications not captured by the database search. Reports were included if one or more patients underwent pancreatic IRE on at least two separate occasions and excluded if only one pancreatic IRE was performed, retreatment was confined to metastatic lesions, repeat pancreatic IRE could not be confirmed, or clinical information was insufficient. For overlapping reports, the report providing the most complete repeat-IRE information was retained to avoid double counting. Six publications involving 25 patients were included; screening is shown in **Supplementary Figure 1**. Kluger et al. reported three patients retreated for local recurrence 7, 9, and 19 months after initial IRE (4). Narayanan et al. reported nine patients undergoing repeat percutaneous IRE for CT-confirmed residual tumor (5). Flak et al. reported six patients; five underwent two IRE procedures and one underwent three, with retreatment offered for isolated local progression when technically feasible (6). PANFIRE-2 included five patients retreated for isolated recurrence within the previous ablation zone (7). Polyakov et al. described one patient retreated after local recurrence (8), and Wall et al. reported one patient undergoing a second percutaneous IRE 14 months after initial open IRE for local progression (9). Given incomplete patient-level data, indications, intervals, techniques, complications, and outcomes were summarized descriptively in **Table 1**.

**Table 1:** Summary of published reports of repeat pancreatic irreversible electroporation.

First author, year	Repeat IRE/overall cohort, n/N	Indication for repeat IRE	Interval between IRE procedures	Repeat IRE approach	Repeat-IRE outcome
Kluger et al. (2016) (4)	3/50	Local recurrence	7, 9, and 19 months	Open, intraoperative US-guided	NR
Narayanan et al. (2017) (5)	9/50	Residual tumor on CT	NR	Percutaneous, CT-guided	NR
Flak et al. (2019) (6)	6/33	Isolated local progression	NR	Percutaneous, US-guided	NR
Ruarus et al. (2020) (7)	5/50	Solitary ablation-zone recurrence	NR	Percutaneous, CT-guided	NR
Polyakov et al. (2022) (8)	1/1	Biopsy-confirmed recurrence adjacent to the ablation zone	Approximately 8 months	Open, intraoperative US-guided	No complications; time to progression >11 months; survival 16 months after repeat IRE
Wall et al. (2022) (9)	1/20	Local progression	Approximately 14 months	Percutaneous, US/CT-guided	NR

CT, computed tomography; IRE, irreversible electroporation; NR, not reported; US, ultrasonography.

Taken together, published experience shows that repeat pancreatic IRE has been used for radiologically confirmed residual tumor after initial IRE, subsequent local recurrence or progression, and isolated recurrence within the previous ablation zone. Among reports providing retreatment intervals, repeat IRE was performed approximately 7–19 months after the initial procedure (4, 8, 9). Although long-term outcomes are infrequently reported and standardized criteria for patient selection and response assessment remain unavailable, these reports support the technical feasibility of repeat IRE in carefully selected patients. Against this background, the present case is distinguished by an interval of nearly 5 years between the two pancreatic IRE procedures. Despite developing liver metastases and receiving multiple systemic and local treatments, the patient had an ECOG performance status of 1, and MDT reassessment indicated that the pancreatic and hepatic segment VI lesions remained technically amenable to local treatment. Repeat pancreatic IRE thus represented a renewed local intervention after prolonged survival with disease, rather than a short-term adjunct to the initial procedure, and was combined with RFA of the hepatic lesion. After the combined local treatment, CA19–9 decreased from 12,280.0 to 472.1 U/mL before adebrelimab was resumed and to 291.4 U/mL thereafter. Early follow-up CT showed no definite residual enhancement, new lesions, or further progression. Together, this course documents the technical feasibility of late repeat pancreatic IRE as part of long-term multimodal management, accompanied by clinically relevant early biochemical and imaging changes.

Survival outcomes from pancreatic IRE studies require contextual interpretation because patient selection, procedural approaches, and subsequent treatment vary. The encouraging observational results reported by Martin et al. may have been influenced by patient selection and immortal-time biases (23, 24). In the randomized phase II CROSSFIRE trial, which enrolled patients pretreated with FOLFIRINOX, no significant difference in median overall survival was observed between IRE and MRI-guided stereotactic ablative body radiotherapy (12.5 vs. 16.1 months; P = 0.21) (27). Because no systemic-therapy-only control group was included, the added value of either local modality over continued systemic therapy remains uncertain. These findings do not establish an independent survival benefit attributable to IRE; instead, they support positioning IRE as a selectively applied local-control component within multimodal care. Case-specific interpretation also requires consideration of the historical treatment context. The 2021 classification as unresectable reflected the assessment that safe reconstruction of the involved SMV-PV segment was not feasible. Under the criteria in NCCN Version 2. 2026, venous reconstructability remains central to resectability assessment; therefore, retrospective interpretation of the initial classification should remain cautious (2). In 2021, obstructive jaundice required surgical management, and the patient proceeded directly to individualized IRE, whereas current consensus recommends induction systemic therapy before IRE to assess tumor biology and optimize patient selection (25). Given the single-case design, concurrent hepatic RFA, postoperative resumption of adebrelimab, and approximately 4 months of follow-up, the observed biochemical and imaging changes are best interpreted as early outcomes of the overall multimodal treatment course.

By extending the interval between pancreatic IRE procedures to nearly 5 years, this case broadens the available experience beyond the shorter retreatment intervals described previously and provides a rare example of MDT-guided repeat local intervention during long-term multimodal management. The clinical course highlights the value of dynamically reassessing local-treatment opportunities when pancreatic disease progresses after initial IRE but remains technically amenable to further intervention. Future studies should refine patient selection and optimal timing, clarify integration with systemic and other local therapies, and evaluate local control, safety, quality of life, and long-term outcomes.

## 4 Conclusion

This case documents the long-term integration of systemic therapy and individualized local interventions under MDT guidance in a patient with initially unresectable LAPC who subsequently developed hepatic metastases and remained alive with disease for more than 62 months. Repeat pancreatic IRE, performed nearly 5 years after the initial procedure together with RFA of a hepatic metastasis, was technically feasible and was followed by a marked decrease in CA19-9, while follow-up imaging showed no definite progression. This case supports the potential role of repeat pancreatic IRE after a prolonged interval as an individualized local-control strategy within long-term multimodal management.

## 5 Patient perspective

When I was first told that my tumor was unresectable, I feared my life was ending. IRE and long-term treatment gave me more time. When my tumor marker later rose markedly, repeat IRE improved my condition again. More than five years later, I am still living with my family and remain deeply grateful to Dr. Chen and his team.

## Data Availability

The original contributions presented in the study are included in the article/**Supplementary Material**. Further inquiries can be directed to the corresponding author.
